# Influence of Temperature and Preserving Agents on the Stability of Cornelian Cherries Anthocyanins

**DOI:** 10.3390/molecules19068177

**Published:** 2014-06-17

**Authors:** Bianca Moldovan, Luminiţa David

**Affiliations:** “Babeş-Bolyai” University, Faculty of Chemistry and Chemical Engineering, 11, Arany Janos Str., 400028 Cluj-Napoca, Romania; E-Mail: bianca@chem.ubbcluj.ro

**Keywords:** anthocyanins, degradation kinetics, potassium sorbate, sodium benzoate

## Abstract

Cornelian cherry (*Cornus mas L.*) fruits are known for their significant amounts of anthocyanins which can be used as natural food colorants. The storage stability of anthocyanins from these fruit extracts, at different temperatures (2 °C, 25 °C and 75 °C), pH 3.02, in the presence of two of the most widely employed food preserving agents (sodium benzoate and potassium sorbate) was investigated. The highest stability was exhibited by the anthocyanin extract stored at 2 °C without any added preservative, with half-life and constant rate values of 1443.8 h and 0.48 × 10^−3^ h^−1^, respectively. The highest value of the degradation rate constant (82.76 × 10^−3^/h) was obtained in the case of anthocyanin extract stored at 75 °C without any added preservative. Experimental results indicate that the storage degradation of anthocyanins followed first-order reaction kinetics under each of the investigated conditions. In aqueous solution, the food preservatives used were found to have a slight influence on the anthocyanins’ stability.

## 1. Introduction

Cornelian cherry (*Cornus* *mas* *L*.) is a species of dogwood native to Southern Europe and Southwest Asia. The fruit is an oblong, red drupe, 2–3 cm long, containing a single seed, edible, but astringent when unripe. Fresh cornelian cherry fruits contain twice as much ascorbic acid (vitamin C) as oranges, being also rich in sugar, organic acids and tannins [1]. Cornelian cherry fruits also contain significant amounts of anthocyanins which are known to possess antioxidant and anti-inflammatory effects. The most popular application of cornelian cherries is in different drinks, gels and jams, but they can also be eaten fresh, dried whole or pickled. The use of Cornelian cherries for the medical treatment of gastrointestinal disorders and diarrhea has been reported [2]. The anti-bacterial, anti-histamine, anti-allergic, anti-microbial and anti-malarial properties of the fruits are also known [3]. In Europe, Cornelian cherry fruits were reported to have food and cosmetic applications [4]. Because of their health benefits, there are several reports about Cornelian cherry fruits, especially regarding their physical and chemical properties, as well as their polyphenolic, ascorbic acid and anthocyanin contents [5].

Anthocyanins are a class of naturally occurring phenols, being the largest group of water-soluble pigments in plants. Many edible plants are sources of anthocyanins [6,7,8]. These compounds play a significant role in the color of many fruits, flowers, vegetables and products derived from them. In recent years, various important biological activities, such as antioxidant, antimutagenic, anticancer, anti-inflammatory and antiobesity properties of anthocyanins have been reported [9,10,11,12]. The bright color of anthocyanins (orange, red, purple, blue), ensures a high potential of being used as natural colorants, as a healthy alternative to synthetic dyes. Color directly affects the appearance and the consumer acceptability of the fruits and their derived products.

**Scheme 1:**
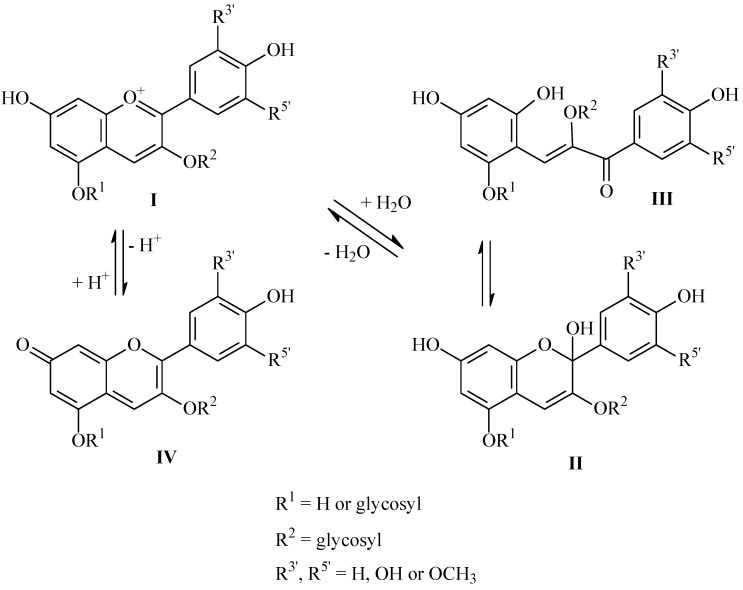
Chemical structures of anthocyanins at different pH values.

The color of anthocyanins depends essentially on the different structural forms in which they can be found, these structures being strongly influenced by the pH value. At pH values between 4 and 6 (typical for fresh and processed fruits) a mixture of equilibrium forms of anthocyanins: red flavylium cation (I), blue anhydrous quinoidal base (IV), colorless carbinol pseudobase (II) and yellow chalcone (III) coexists (Scheme 1).

Anthocyanins easily convert to undesirable colorless or brown compounds as a consequence of their high reactivity. The anthocyanin stability can be influenced by many factors, the most important being temperature. The light, pH value, presence of oxygen, ascorbic acid, sugars, hydrogen peroxide and enzymes also affect the stability of these natural pigments [13,14,15,16,17,18].

Thus, investigation of anthocyanins degradation and measurement of their content at various intervals of storage offers useful experimental information for the food industry. However, to date, no information is available in the literature on the degradation kinetics of Cornelian cherry anthocyanins. The accurate determination of the degradation kinetics for these compounds during storage or thermal processing is essential for predicting changes that may occur in food products containing these anthocyanins.

Sodium benzoate and potassium sorbate are used as food preservatives due to their antimicrobial properties. They are widely used in foods such as soft drinks, jams and fruit juices. Although the effect of these compounds on the inactivation of bacterial pathogens in fruit juices was reported [19], the influence of these synthetic food preserving agents on anthocyanin degradation has been little investigated. Thakur and Araya tested the effect of sodium benzoate and sorbate on the stability of blue grape anthocyanins during storage at 15–35 °C [20], indicating that the stability of the anthocyanins after 60 days was higher in samples preserved with sorbate than with benzoate.

The objective of the present study was to evaluate the influence of temperature and these two commonly used synthetic food preservatives on the stability of Cornelian cherry anthocyanins. The investigated conditions (temperature and nature of an added preservative) ensure a high compatibility with processing techniques often applied in the food industry.

## 2. Results and Discussion

The influence of temperature and food preserving agents on the stability of anthocyanins from the Cornelian cherry fruits extract during storage was investigated. The determined values for the kinetic parameters (kinetic rate constants and the half-life values) are summarized in Table 1.

**Table 1:** Kinetic parameters of degradation of anthocyanins from Cornelian cherriesextracts in different conditions.

Sample	Temp. (°C)	k∙10^−3^ (h^−1^) ^1^	t_1/2_ (h) ^2^
Crude extract	2	0.48 (0.9632)	1443.75 ^a^
Extract+sodium benzoate	2	0.57 (0.9733)	1215.78 ^b^
Extract+potassium sorbate	2	0.65 (0.9903)	1066.15 ^c^
Crude extract	22	0.87 (0.9188)	796.55 ^a^
Extract+sodium benzoate	22	0.93 (0.9739)	745.16 ^b^
Extract+potassium sorbate	22	1.13 (0.9703)	613.27 ^c^
Crude extract	75	82.61 (0.9922)	8.38 ^a^
Extract+sodium benzoate	75	78.84 (0.9908)	8.78 ^a^
Extract+potassium sorbate	75	77.35 (0.9912)	8.95 ^a^

^1^ Numbers in parentheses, R^2^, are the determination coefficients; ^2^ Values within a column with different superscript letters are significantly (*p* < 0.05) different in the same temperature group.

The content of anthocyanins from Cornelian cherries aqueous extract during storage at different temperatures (2 °C, 22 °C and 75 °C) were plotted as a function of time. The initial total content of the extract was 68.68 ± 0.088 mg/L. The linear regression of the total anthocyanins content of Cornelian cherry fruits extracts during storage confirmed that the degradation process of these pigments followed first order reaction kinetics. These results are in agreement with previously reported literature data [15,21,22,23,24] that indicated first order reaction kinetics for the storage and thermal degradation of anthocyanins from various sources.

The thermal stability of the extracts was evaluated. As expected, the increase of storage temperature (22 °C) resulted in a 1.8 times faster degradation as compared to refrigerated storage (at 2 °C) while, at 75 °C, the degradation rate was 172.1 times higher.

**Figure 1:**
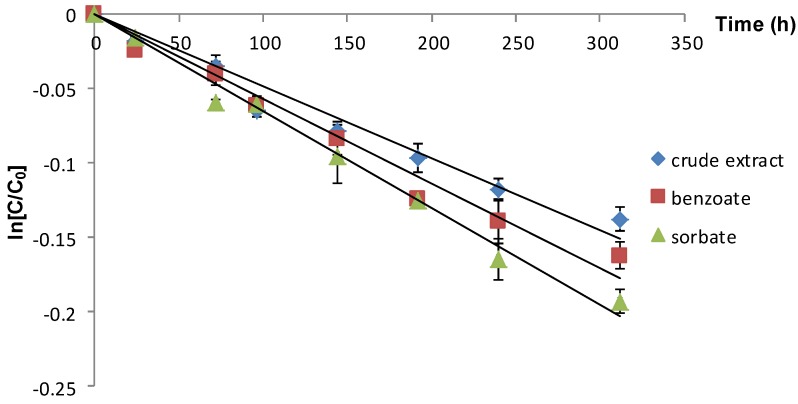
Influence of different food preservatives on the anthocyanin stability during storage at 2 °C (vertical lines represent SD, *n* = 4).

The total content of anthocyanins from Cornelian cherries extracts stored at 2 °C was plotted as a function of time (Figure 1). By comparing the half-life values, one can conclude that, the presence of food preservatives displayed a slightly destabilizing effect on the anthocyanins from the investigated extracts.

However, the difference between the two added food preservatives was not significant, the degradation process being 1.14 fold faster in the presence of potassium sorbate as compared to sodium benzoate.

**Figure 2:**
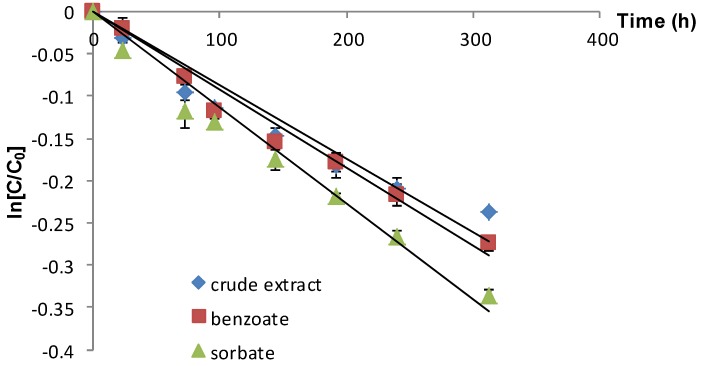
Influence of different food preservatives on the anthocyanin stability during storage at 22 °C (vertical lines represent SD, *n* = 4).

As observed in the case of refrigerated storage, the Cornelian cherry anthocyanins stored at 22 °C showed the same degradation profile (Figure 2). In this case, storage of the extracts in the presence of potassium sorbate resulted in faster degradation compared to storage with added sodium benzoate, the value of the half-life ratio being 1.21.

**Figure 3:**
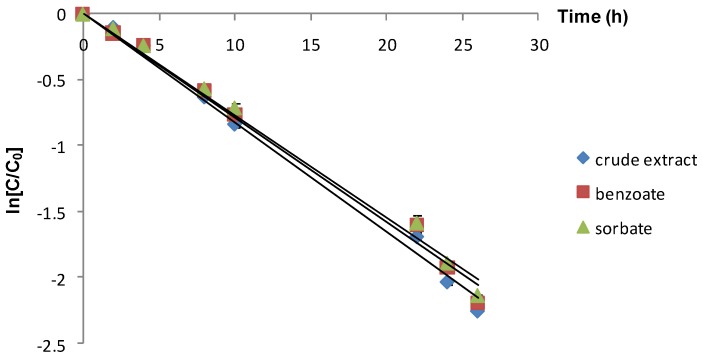
Influence of different food preservatives on the anthocyanin stability during storage at 75 °C (vertical lines represent SD, *n* = 4).

As expected, the increase of temperature at 75 °C resulted in an accelerated degradation of anthocyanins. During high temperature storage, as applied at 75 °C, the destabilizing effect of sodium benzoate and potassium sorbate on the anthocyanin pigments (observed at lower storage temperatures) was not evident. In contrast, the added food preserving agents had almost no influence on the stability of these pigments (Figure 3), the half-life values being practically the same for all the investigated extracts (8.4 ÷ 8.95 h).

The effect of temperature on the kinetics of the degradation process was determined by fitting the rate constants to an Arrhenius type equation.

**Figure 4:**
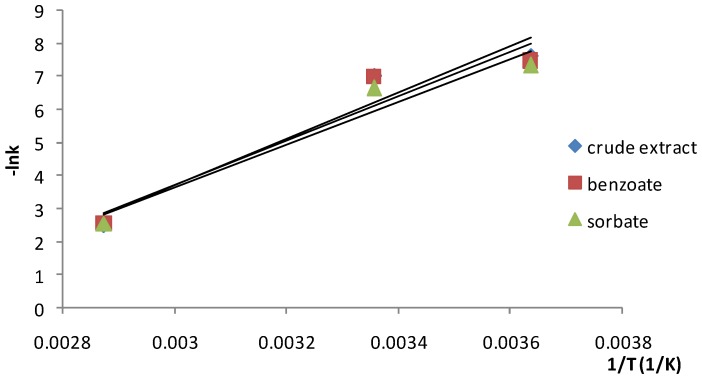
The Arrhenius plots for degradation of anthocyanins in Cornelian cherries extracts.

The anthocyanin degradation rate constants obtained for each extract were plotted as a function of temperature (Figure 4).

The calculated activation energies (E_a_), frequency factors (K_o_) and the temperature coefficients (Q_10_) are given in Table 2.

**Table 2:** Effect of temperature on the degradation of anthocyanins from Cornelian cherry fruits extracts.

Solvent	E_a_ (kJ/mol) ^a^	K_o_ (h^−1^)	Q_10_	
			2–22 °C	22–75 °C
Crude extract	58.55 (0.9307)	3.72 × 10^7^	1.346	2.361
Extract+sodium benzoate	56.21 (0.9226)	1.57 × 10^7^	1.277	2.311
Extract+potassium sorbate	54.09 (0.9446)	7.9 × 10^6^	1.318	2.219

^a^ Numbers in parentheses, R^2^, are the determination coefficients.

Since high activation energy reactions are more sensitive to temperature, the anthocyanins in the extract proved to be more susceptible to degradation by exposure to elevated temperatures. The calculated E_a_ values ranged from 54.09 to 58.55 kJ/mol. The highest influence of the temperature on the stability of the investigated compounds (the highest value of E_a_) was observed for the anthocyanins stored in the crude extract, while the pigments stored in the presence of potassium sorbate exhibited lower susceptibility to thermal degradation, presenting the lowest value of the E_a_. The low differences between the activation energy values suggested that the added synthetic preservatives exhibited a slightly influence on the stability of anthocyanins.

In order to evaluate the dependence of degradation rate on temperature, the temperature coefficient Q_10_ (the change of degradation rate upon a temperature increase of 10 K) was calculated.

Higher Q_10_ values for storage temperatures of 22–75 °C were obtained, indicating that anthocyanins are more sensitive to temperature elevations at high storage temperatures compared to low storage temperatures (2–22 °C) where the Q_10_ values were ranged from 1.277 to 1.346, whereas the differences were insignificant. The lowest temperature coefficient value (1.277 at 2–22 °C) was obtained for the anthocyanins stored in the presence of sodium benzoate. Almost the same Q_10_ values were obtained for the degradation of anthocyanins stored at 2–22 °C for all the investigated extracts (crude or with added preservative), proving that the influence of the added food preserving agents was not significant. Storage at 22–75 °C resulted in higher Q_10_ values the calculated temperature coefficients for this storage interval presenting almost the same value for all the investigated extracts. Higher Q_10_ values indicate that at high storage temperatures (22–75 °C) anthocyanins are more sensitive to temperature elevations than at low storage temperatures (2–22 °C).

All these results clearly indicate that low storage temperatures are required to inhibit the degradation process of these pigments from Cornelian cherry extracts.

Significantly different temperature coefficients Q_10_ for the two investigated temperature intervals, may be due to a possible change in the reaction mechanism of the degradation of Cornelian cherries anthocyanins at elevated temperatures, such as 75 °C, compared to low temperature degradation process. The high ascorbic acid content of Cornelian cherry fruits [1] could accelerate the degradation of anthocyanins. The loss of anthocyanins caused by ascorbic acid (AA) occurs due to the free radical oxidative cleavage of the pyrilium ring in which AA acts as molecular oxygen activator. At high temperatures, AA itself undergoes a degradation process, generating degradation products which are also responsible for anthocyanins degradation [25]. Since no degradation studies were performed on the Cornelian cherry anthocyanins, the determined t_1/2_ values are compared to the literature reported data for the degradation process of anthocyanins obtained from other fruits. Wang and Xu reported that the t_1/2_ value for anthocyanin degradation in blackberry juice at pH 2.86 was 4.7 h at 80 °C [23]. In blood orange juice concentrate, the reported t_1/2_ value at 4 °C was 55.7 days [26]. Compared to this value, our results are in the same range (t_1/2_ = 60.2 days at 2 °C). The major anthocyanins in Cornelian cherries are cyanidin-3-*O*-galactoside, pelargonidin-3-*O*-galactoside and delphinidin-3-*O*-galactoside [3]. However, the major anthocyanins in blackberry are cyanidin-3-*O*-glucoside, cyanidin-3-*O*-rutinoside and cyanidin-3-*O*-malonyl-glucoside [27,28]. Therefore, the different stability of the anthocyanins might be due to the varying composition of the fruit extracts; the major constituents of these extracts being sugars, ascorbic acid, and flavonoids, which are known to be intrinsic factors that influence anthocyanins degradation [29]. It could be concluded that Cornelian cherry anthocyanins are more stable than anthocyanins from other sources (e.g., blackberry), proving a quite good stability during storage and heating, indicating a potential use of these pigments as natural colorants in food industry.

## 3. Experimental

### 3.1. Materials

#### 3.1.1. Plant Material

Samples of Cornelian cherry fruits were purchased in August 2012 from a local market in Cluj-Napoca, Romania. Fruits were packed in polyethylene bags and kept frozen at −18 °C before being subjected to extraction of anthocyanins.

#### 3.1.2. Chemicals and Reagents

Potassium chloride, sodium acetate, acetic acid and HCl conc., were purchased from Merck (Darmstadt, Germany). Sodium benzoate and potassium sorbate were purchased from Chimopar (Bucharest, Romania). All chemicals and reagents were of analytical grade and were used without further purification. The distilled water was obtained using a TYPDP1500 Water distiller (Techosklo LTD, Držkov, Czech Republic).

### 3.2. Methods

#### 3.2.1. Preparation of Anthocyanin Extract

Fifty grams of frozen Cornelian cherry fruits were crushed in a mortar. Thirty five g of fruit puree were transferred to an Erlenmeyer flask and distilled water (200 mL) and concentrated HCl (0.25 mL) were added. The mixture was stirred for 1 h at room temperature and then filtered. The residue was washed twice with extraction solvent (acidified water, 20 mL). The filtrate was quantitatively transferred to a 250 mL volumetric flask and made up to 250 mL with solvent. The pH of the extract was determined using a Hanna Instruments (HI) 99161 pH-meter and the measured value was 3.02.

#### 3.2.2. Determination of Anthocyanin Content

The total anthocyanin content was determined using optical spectroscopy, by the convenient method of Giusti and Wrolstad [30]. This method is based on the structural changes of the pigments as a function of pH. At pH = 1, the red to purple oxonium form predominates while at pH = 4.5 the major structural form is the colourless hemiketal. The difference in absorbance of the anthocyanin solutions at these two pH values permits an accurate and rapid determination of total monomeric anthocyanin content in the sample matrix. The two desired pH values were reached using two buffer systems: potassium chloride buffer (0.025 M, pH = 1.0) and sodium acetate buffer (0.04 M; pH = 4.5).

Aliquots of Cornelian cherry fruits extract (5 mL) were transferred to a 10 mL volumetric flask, made up to 10 mL with corresponding buffer (pH = 1 and pH = 4.5) and allowed to equilibrate for 15 min. The absorbance of each equilibrated solution was then measured at 506 (the wavelength where the maximum of absorbance occurs = λ_VIS_ _max_) and 700 nm (for haze correction), using an UV-VIS Perkin Elmer Lambda 25 double beam spectrophotometer.

Pigment content was calculated using a molar extinction coefficient of 26,900 L/mol∙cm and a molecular weight of 449.2 g/mol (cyanidin-3-glucoside). Results were expressed as mg cyanidin-3-glucoside equivalents∙L^−1^ extract. Visible spectra of samples were recorded by scanning the absorbance between 400 and 700 nm. Quartz cuvettes of 1 cm path length were used. Absorbance readings were performed against distilled water as a blank. All the measurements were carried out at room temperature (~22 °C).

The total anthocyanin content (expressed as cyanidin-3-glucoside equivalents), was calculated from the experimental data, using the following equation [30]:

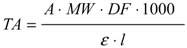
(1)
where: TA = total anthocyanin content (mg/L) ; A = absorbance, calculated as: [Equation (6)]

A = (A_pH__1.0_ − A_pH__4.5_) _506__nm_ − (A_pH__1.0_ – A_pH__4.5_) _700__nm_ (2)

MW = molecular weight; DF = dilution factor; l = path length; ε = molar extinction coefficient; 1000 = conversion factor from grams to milligrams.

Four determinations (*n* = 4) were performed for each analysis and the average values of total anthocyanin content were used for kinetic parameters determination.

#### 3.2.3. Degradation Studies

The influence of temperature on the storage stability was studied at 2 °C, 22 °C and 75 °C. Each extract was divided into 50 mL portions and kept away from light (well capped to avoid evaporation) at 2 °C (in refrigerator), at room temperature (22 °C) and in a thermostatic water bath, preheated to 75 °C, respectively (±1 °C).

To test the influence of food preservatives on the thermal stability of anthocyanins from Cornelian cherry fruits extract, sodium benzoate or potassium sorbate was dissolved in the fruits extract at a final concentration of 1 g/L. 

Changes in total anthocyanin content of the samples were measured in order to evaluate the stability of the pigments in the investigated extracts. Samples were analyzed at 0, 1, 3, 4, 6, 8, 10 and 13 days for all extracts except for those stored at 75 °C, which were sampled at 0, 2, 4, 8, 10, 22, 24 and 26 h. The storage intervals were different for the last thermal treatment due to the differences in anthocyanin degradation rates.

#### 3.2.4. Degradation Kinetics

The kinetics for the degradation reaction of the investigated anthocyanins can be expressed by the equations:

ln[TA] = ln[TA_0_] − kt (3)

t_1/2_ = −ln0.5/k (4)

where: [TA] = total anthocyanin content (mg/L) at time t; [TA_0_] = initial total anthocyanin content (mg/L); k = reaction rate constant (h^−1^); t = reaction time (h); t_1/2_ = half−life (h).

The effect of temperature on the kinetics of the degradation process was determined by fitting the rate constants to an Arrhenius type equation [Equation (5)]:

k = K_0_e^−Ea/RT^ (5)

where: k = rate constant (h^−1^); K_0_ = frequency factor (h^−1^); E_a_ = activation energy (kJ/mol); R = universal gas constant (8.314 J/mol∙K); T = absolute temperature (K).

The Q_10_ temperature coefficient was calculated according to Equation (6):

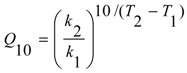
(6)
where: Q_10_ = the temperature coefficient (K^−1^); k_1,2_ = rate constant (h^−1^) at temperature T_1,2_ (K).

### 3.3. Statistical Analysis

Data are reported as mean values of at least four experiments. Results were analyzed using one-way variance analysis (ANOVA). Analysis of variance was performed using XLSTAT Release 10 (Addinsoft, Paris, France). Differences at *p* < 0.05 were considered statistically significant.

## 4. Conclusions

The total anthocyanin content of Cornelian cherries and the storage stability of these compounds indicated that these fruits can be used as an important source of natural red pigment for the food industry. The results of the present study have provided detailed information on the degradation kinetic parameters of anthocyanins during storage and heating. Increasing the temperature resulted in higher degradation rate constants: the degradation rate of anthocyanins from Cornelian cherries extract at 22 °C was 1.8 times faster than at 2 °C, while at 75 °C this process was 172 times faster than at 2 °C. Comparison of the rate constants and half-life values showed that the anthocyanin stability was slightly influenced by the kind of the added organic food preservative.
